# From Cellulose to Functional Electrode SCNF:rGO Hybrid Films for Electrochemical Applications

**DOI:** 10.3390/polym17233225

**Published:** 2025-12-04

**Authors:** Josefa Silva, José Raúl Sosa-Acosta, Galo Ramírez, Katherina Fernández, Rodrigo del Rio

**Affiliations:** 1 Laboratorio de Biomateriales, Departamento de Ingeniería Química, Facultad de Ingeniería, Universidad de Concepción, Concepción 4030000, Chile; jossilva2017@udec.cl; 2 Departamento de Química Inorgánica, Facultad de Química, Pontificia Universidad Católica de Chile, Av. Vicuña Mackenna 4860, Macul, Casilla 306, Correo 22, Santiago 7820436, Chile; jrsosa@uc.cl (J.R.S.-A.); gramirezj@uc.cl (G.R.); 3 Millennium Institute on Green Ammonia as Energy Vector (MIGA), Av. Vicuña Mackenna 4860, Macul, Casilla 306, Correo 22, Santiago 7820436, Chile

**Keywords:** electrochemical sensor, reduced graphene oxide, sulfated nanocellulose, hydrazine electrooxidation, electrode material

## Abstract

Sulfated nanocellulose (SCNF) and reduced graphene oxide (rGO) films were fabricated through environmentally friendly methods to develop an effective platform for electrochemical applications. The hybrid materials were extensively characterized by FTIR, XRD, Raman spectroscopy, TGA, SEM, cyclic voltammetry (CV), and electrochemical impedance spectroscopy (EIS). Results showed that incorporating rGO into the SCNF matrix significantly improved the electrical conductivity and structural robustness of the films. FTIR confirmed interactions between sulfate groups on cellulose and residual oxygen-containing groups on rGO, while XRD and Raman analyses indicated reduced crystallinity and increased structural disorder, supporting the successful integration of both phases. XPS further demonstrated that SCNF and rGO form chemical bonds rather than simply mixing, with both components remaining active at the surface—evidence of strong interfacial interactions that contribute to enhanced stability and efficient charge transfer. The 1:5 (rGO:SCNF) composition showed the best electrochemical performance, exhibiting minimal charge-transfer resistance and improved hydrazine oxidation, as reflected by a shift of the anodic peak potential toward lower values. Additionally, functionalization with cobalt porphyrin significantly boosted catalytic activity. Overall, the SCNF:rGO films offer a sustainable and scalable platform for electrochemical sensing and energy-conversion applications, demonstrating excellent adaptability and functional performance.

## 1. Introduction

The replacement of conventional materials has heightened interest in natural biopolymers. These compounds offer an attractive balance between functionality, low environmental impact, and availability, making them essential for driving advances in green technologies [1]. Cellulose, widely recognized as the most abundant natural polymer on Earth [2], has gained prominence as a strategic raw material to address the challenges of the energy transition due to its availability, low cost, and biodegradability [3,4]. Through various refining or depolymerization processes, cellulose can be transformed into nanomaterials with enhanced physicochemical properties. These nanomaterials are primarily classified into cellulose nanocrystals (CNC), characterized by a rigid, highly crystalline structure, and cellulose nanofibrils (CNF), produced via mechanical treatments that generate flexible nanometric networks [5,6,7].

A functional variant of CNF is sulfated nanocellulose (SCNF), obtained by chemical sulfation in an acidic medium. Traditionally, this process employs sulfuric acid, which introduces sulfate groups onto the fiber surface, increasing negative charge and colloidal stability. However, Taru Negi [8] and Xinyu Wu [9] propose the use of deep eutectic solvents (DES) as an eco-friendly and safe alternative for this procedure. In this study, a mixture of sulfamic acid and urea was used to induce direct sulfation without organic solvents, thereby facilitating the efficient incorporation of sulfate groups (–SO_3_^−^) onto fiber surfaces. Unlike previously reported DES-based sulfation approaches—which generally operate under milder conditions (<120 °C) and focus mainly on cellulose functionalization—our method employs a controlled thermal activation step (150 °C, 30 min) that enhances sulfate grafting and increases surface charge density. This higher charge favors stronger interfacial interactions with reduced graphene oxide (rGO), enabling uniform hybrid formation and extending DES chemistry beyond simple cellulose modification and enhancing interactions with conductive polymers and metallic materials.

Additionally, reduced graphene oxide (rGO), derived from graphene via chemical reduction, exhibits high electrical conductivity, a large surface area, and good compatibility with composite matrices [10,11]. Öztekin’s study [12] indicates that by partially restoring the conjugated carbon network in oxidized graphene, rGO becomes an effective medium for electron transport. Similarly, Mellado and Figueroa [13] demonstrated that ultrasonication treatments—either via probe or bath—significantly influence the material’s structure, residual functional groups, degree of exfoliation, and final morphology, all of which directly impact its electrochemical behavior. However, to exploit these properties effectively, a matrix that facilitates dispersion and anchoring is required. In this context, SCNF offers a sustainable solution: its high surface charge and good dispersion in aqueous media promote the formation of homogeneous hybrid films [14], combining the structural properties of nanocellulose with the conductivity of rGO.

These hybrid platforms are relevant for energy applications, particularly in devices employing electrochemical processes as means of energy conversion. One key reaction in this field is electrooxidation, whereby a chemical species loses electrons at the electrode surface upon applying an electrical potential [15]. In this context, hydrazine (N_2_H_4_) has been highlighted as a promising energy carrier in direct fuel cells, due to its high energy density and the formation of non-contaminant products such as molecular nitrogen [16].

Furthermore, the electrooxidation of hydrazine has gained prominence in electrochemical sensing, as it exhibits a well-defined anodic process whose efficiency depends on the electrode’s surface properties [17]. Nonetheless, this reaction exhibits slow kinetics on conventional electrodes, prompting the development of modified materials that enhance charge transfer and reduce overpotential. Recent studies have explored various materials to improve hydrazine electrooxidation efficiency. Crisalli [18] developed electrodes modified with Ni-Co nanoparticles supported on carbon, achieving good catalytic activity under alkaline conditions. Additionally, Canales and Ramírez [19] reported a modification of a glassy carbon electrode (GCE) with metalated octaethylporphyrins (M-OEP) for hydrazine electrooxidation in neutral media, representing an efficient candidate for the design of sensitive electrochemical sensors. However, this approach involves complex materials, which may be challenging to produce and stabilize for large-scale sustainable applications. Therefore, it is proposed to synthesize a metal-free hybrid film composed solely of sulfated nanocellulose (SCNF) and reduced graphene oxide (rGO), evaluating their morphological and functional properties and their electrochemical behavior toward hydrazine oxidation. The electrochemical performance of the hybrid films will be assessed through cyclic voltammetry (CV) and electrochemical impedance spectroscopy (EIS), analyzing their behavior as electrode materials considering stability in aqueous media and potential application in energy storage devices. This platform requires no prior electrode functionalization and acts as a self-supporting system that combines biocompatibility, porosity, colloidal stability, and high conductivity within a continuous phase. The novelty of this research lies in developing a hybrid film entirely from plant-derived materials using environmentally friendly processes, such as DES. This approach reduces the environmental impact of manufacturing and provides a functional, dispersible platform in aqueous media with a high surface area, opening new possibilities for green sensor design and clean energy conversion technologies.

## 2. Materials and Methods

Bleached eucalyptus Kraft pulp was supplied by Arauco S.A. (Santiago, Chile). The deep eutectic solvent (DES) was prepared using sulfamic acid (H_3_SO_3_NH_2_, ≥98%) and urea (CH_4_N_2_O; Winkler, Santiago, Chile). Sulfuric acid (H_2_SO_4_, 97–99%), phosphoric acid (H_3_PO_4_, 85%), hydrochloric acid (HCl, 10% *v*/*v*), TRIS buffer, dopamine, 2-propanol, and hydrazine sulfate (N_2_H_6_SO_4_) were obtained from Sigma-Aldrich (Oakville, ON, Canada). Activated carbon (Arquimed, Santiago, Chile) was used as the carbon precursor for the synthesis of graphene oxide (GO) and reduced graphene oxide (rGO) via a modified Hummers method, employing potassium permanganate (KMnO_4_) as the oxidizing agent and hydrogen peroxide (H_2_O_2_, 50% *w*/*w*) as the quenching agent. The resulting material was purified by HCl washing and confirmed by AgNO_3_ addition, followed by dialysis (12 kDa) in Milli-Q water. For electrochemical measurements, hydrazine sulfate served as the model analyte and supporting electrolyte, while 2-propanol was used as the dispersing agent for rGO. A glassy carbon electrode (GC, Ø3 mm) was used as the working electrode, with an Ag/AgCl electrode as the reference and a graphite rod as the counter electrode (Gamry Instruments, Warminster, PA, USA).

### 2.1. Obtention of Sulfated Nanocellulose (SCNF) Using DES

The SCNF were obtained using a DES composed of sulfamic acid (H_3_NSO_3_) and urea (CH_4_N_2_O) in a 1:3 molar ratio. The mixture was gradually heated to 80 °C under constant stirring, yielding a homogeneous and transparent solution, into which dry cellulose pulp (BKP) was incorporated. Sulfation was carried out under continuous stirring, increasing the temperature to 150 °C for 30 min, which promoted the incorporation of sulfate groups onto the fiber surface.

At the end of the reaction, the mixture was quenched with distilled water, and the fibers were separated by filtration, followed by successive washing steps to remove residual acid and urea. Finally, the sulfated pulp was hydrated with distilled water to reach a concentration of 1% (*w*/*w*) and mechanically homogenized through industrial filtration, resulting in a stable colloidal dispersion of SCNF. To evaluate the reproducibility of the DES-based sulfation process, several independent batches were prepared under identical reaction conditions. The freeze-dried SCNF obtained from each batch was analyzed by FTIR, showing only minor variations in the relative intensity of the sulfate-associated bands, which confirms a consistent degree of sulfation. Likewise, SEM imaging of replicated batches revealed no significant changes in fiber morphology: all samples displayed the characteristic fibrillar network of sulfated cellulose, without evidence of over-sulfation, structural collapse, or excessive fragmentation. These observations indicate that the DES process yields a stable and reproducible sulfation outcome.

Although the DES route offers several practical advantages over conventional mineral-acid sulfation, we acknowledge that a quantitative assessment of green metrics (e.g., E-factor, atom economy, or solvent-recyclability efficiency) was not performed in the present study. Thus, our description of the process as “environmentally benign” refers primarily to qualitative aspects: the use of a low-toxicity, non-volatile DES instead of strong acids, the absence of metal catalysts or organic reagents, and the practical possibility of reusing the DES phase after simple filtration. A more detailed green-metrics or life-cycle analysis will be addressed in future work.

**Figure 1:**
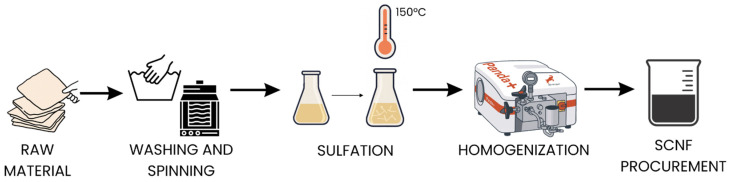
Schematic diagram of the process for obtaining sulfated nanocellulose (SCNF) using deep eutectic solvent (DES).

The overall process for obtaining SCNF using DES is schematically illustrated in Figure 1.

### 2.2. Synthesis and Reduction of Graphene Oxide

Graphene oxide (GO) was synthesized in the laboratory using the modified Hummers method [20]. In a 500 mL beaker, 30 mL of H_3_PO_4_ and 270 mL of H_2_SO_4_ were mixed, followed by the addition of 2.25 g of graphite and subsequently 13 g of KMnO_4_. The reaction was maintained between 40–45 °C for 2 h. After cooling in an ice bath, 55 mL of H_2_O_2_ (60 vol.) was slowly added, resulting in the characteristic color change. The suspension was centrifuged at 5000 rpm for 20 min at 15 °C, and the resulting precipitate was subjected to successive washing steps with 10% *v/v* HCl (as the first wash), followed by Milli-Q water until no ethanol residues remained. The purified material was dialyzed for 3 days using 12 kDa membranes with daily water replacement and finally lyophilized at −40 °C for 3 days, yielding a brown solid corresponding to graphene oxide.

The GO reduction was carried out in aqueous medium using TRIS and dopamine as reducing agents. For this purpose, 400 mL of water containing 0.48 g of TRIS was prepared, and the pH was adjusted to 9 with HCl. Dopamine was then added until reaching a pH of 8.5, after which graphene oxide was incorporated. The mixture was vigorously stirred and sonicated for 30 min to improve homogeneity. The reaction mixture was maintained under heating for 24 h, then filtered and thoroughly washed, followed by dialysis for 3 days to remove impurities.

### 2.3. Preparation of Hybrid Films of Sulfated Nanocellulose (SCNF) and Reduced Graphene Oxide (rGO)

Hybrid films of SCNF and rGO were prepared from a prior dispersion of rGO in 2-propanol. For this purpose, the rGO was sonicated in an ultrasonic bath for 1 h 30 min to ensure a homogeneous dispersion, after which SCNF was incorporated under mechanical stirring at 500 rpm for 4 h. The mixture was then kept under gentle stirring overnight. In cases where aggregates were observed, the suspension was filtered through a 300 µm mesh screen. The final suspension was poured into clean Petri dishes, evenly distributed, and dried in an incubator at 20 °C for 2–3 h until complete solvent evaporation, avoiding bubble formation or deformation. The different formulations used for the preparation of the hybrid films are summarized in Table 1.

Film thickness was controlled by casting a fixed volume of the SCNF:rGO dispersion into identical Petri dishes and drying under constant temperature, time, and ventilation conditions. This ensured that all films were produced with comparable thickness and minimized variability associated with solvent evaporation. The quality of rGO dispersion was maintained through controlled sonication parameters (time and amplitude), followed by visual inspection to confirm the absence of visible aggregates prior to casting. After film formation, the homogeneity of rGO distribution within the SCNF network was assessed by SEM imaging, which revealed uniform surface morphology without micro-scale agglomerates. In addition, the reproducible electrochemical response (CV and EIS) of the 1:5 films served as an indirect indicator of proper rGO dispersion, since poorly dispersed systems typically show higher charge-transfer resistance and unstable voltammetric profiles.

Two rGO:SCNF formulations (1:5 and 2:3) were initially evaluated during the film-forming trials. The 2:3 composition produced fragile and poorly cohesive films that frequently fractured during demolding, indicating that higher rGO loadings compromise the structural integrity provided by the hydrogen-bonded SCNF network. In contrast, the 1:5 formulation yielded homogeneous, flexible, and self-supporting films with no visible defects. For this reason, the 1:5 ratio was selected for subsequent characterization, as it provided the best balance between mechanical stability, rGO dispersion, and reliable film formation.

### 2.4. Film Characterization

#### 2.4.1. Macroscopic Images

The films obtained from SCNF and SCNF:rGO were photographed using a digital camera under controlled lighting conditions and a neutral background to compare color differences, homogeneity, and overall appearance among the samples.

#### 2.4.2. Microscopic Images

The surface morphology of the SCNF:rGO hybrid films was characterized using scanning electron microscopy (ZEISS, Gemini 360, Carl Zeiss, Oberkochen, Germany), operated at an acceleration voltage of 2.0 kV and a magnification of 1.5K. Prior to analysis, the samples were coated with a thin layer of gold to enhance conductivity and improve image resolution and quality.

#### 2.4.3. Fourier Transform Infrared Spectroscopy (FTIR)

Infrared spectra were recorded using an FTIR spectrophotometer (Nicolet iS5, Thermo Scientific, Waltham, MA, USA) in the range of 400–4000 cm^−1^. This analysis allowed the identification of characteristic functional groups of sulfated nanocellulose, as well as the evaluation of possible chemical interactions in the hybrid films containing rGO.

#### 2.4.4. X-Ray Diffraction (XRD)

The crystalline structure of the samples was analyzed by X-ray diffraction using a Bruker^®^ D4 Endeavor diffractometer (Bruker AXS, Karlsruhe, Germany) (Cu Kα radiation, Ni Kβ filter, λ = 1.5406 Å). Diffractograms were collected in the 2θ range of X–Y°, with a step size of 0.02°, operating at 40 kV and 20 mA.

#### 2.4.5. Raman Spectroscopy

The vibrational analysis of the films was carried out using Raman spectroscopy with a LabRAM HR Evolution spectrometer (HORIBA JOBIN YVON, Longjumeau, France) coupled to an optical microscope and controlled by LabSpec 6.4.3 software (HORIBA). A 633 nm excitation laser with a power of 100 mW was used on the sample surface. Spectra were recorded in the range of 500–2000 cm^−1^ with multiple accumulations to identify the D and G bands associated with rGO, as well as possible shifts, broadenings, and intensity variations related to interactions among rGO, the SCNF matrix, and porphyrin. Each Raman spectrum was deconvoluted using Lorentzian-type curves corresponding to the first-order Raman modes: D*, D, D″, G, and D′.

#### 2.4.6. XPS Analysis

The surface chemical characterization of the films was performed using X-ray photoelectron spectroscopy (XPS) with a FlexPS system (SPECS) equipped with a PHOBIOS 150 hemispherical analyzer and a 1D-DLD detector (Berlin, Germany). A FOCUS 500 monochromatic X-ray source providing Al Kα radiation with a characteristic energy of 1486.71 eV was used. This technique allowed the identification of the elements present on the surface and the analysis of their oxidation states, providing detailed information about the chemical composition and possible interactions between the species within the hybrid film.

#### 2.4.7. Thermogravimetric Analysis (TGA)

The thermal stability of the films was evaluated using thermogravimetric analysis (Netzsch STA 409 PC, Selb, Germany), recording mass loss as a function of temperature from 30 to 800 °C. The test was performed at a heating rate of 20 °C/min under a nitrogen atmosphere.

#### 2.4.8. Electrochemical Tests

Electrochemical experiments were performed using a Gamry Interface 1010E potentiostat (Gamry Instruments, Warminster, PA, USA) controlled by Gamry Framework software (version 7.3, Gamry Instruments, Warminster, PA, USA). A conventional three-electrode system was used, consisting of a glassy carbon (GC) working electrode (modified as indicated), an Ag/AgCl reference electrode, and a graphite counter electrode. Electrochemical responses were evaluated in two aqueous solutions: 0.1 M K_3_Fe (CN)_6_ in 1 M KNO_3_, and 5.0 mM hydrazine sulfate in phosphate buffer (pH 7.0). Cyclic voltammetry (CV) measurements were recorded within a potential window of 0 V to +1.2 V at different scan rates, while electrochemical impedance spectroscopy (EIS) was conducted in the frequency range of 100 kHz to 0.01 Hz, using an AC perturbation of 10 mV at the equilibrium potential. The electrodes studied were prepared from suspensions of SCNF and SCNF:rGO in 2-propanol. For this, 1 mL of the dispersion was deposited onto the GC electrode and dried in a BIOBASE incubator (model BJPX-B25011, Jinan, China) at 25 °C with air circulation. A final modification was carried out by depositing 100 µL of a 5 mM cobalt octaethylporphyrin solution in dichloromethane on top of the SCNF:rGO film and allowing it to dry at room temperature.

The electroactive area was calculated using cyclic voltammetry (CV) with a 0.1 M potassium ferricyanide solution as the redox probe. Voltammograms were recorded for both bare GC and GC modified with rGO:SCNF hybrid films. The active area was determined using the Randles–Ševčík equation. Figure S2 (Supplementary Material) shows the voltammograms and corresponding plots used to determine the electroactive area for both electrodes. EIS was subsequently performed at the equilibrium potential with an AC perturbation of 10 mV in the frequency range from 10 mHz to 10 kHz. Figure S3 presents the Nyquist plots of the different electrodes prepared.

#### 2.4.9. Statistical Analysis

The experimental data obtained from the electrochemical measurements were analyzed using descriptive and inferential statistical methods to ensure accuracy and reproducibility. Each condition was evaluated using ten data points, with each measurement recorded two to three times to minimize experimental error. The results are reported as mean ± standard deviation. Data normality and variance homogeneity were verified prior to comparative analyses, and statistical significance was considered at *p* < 0.05. All statistical analyses were performed using Statgraphics 19, Centurion XIX^®^ software.

## 3. Results and Discussion

This section presents the results obtained for the synthesis and characterization of the SCNF:rGO hybrid films. The analysis is structured progressively, beginning with macroscopic and microscopic observations, followed by structural characterization using FTIR, XRD, and Raman spectroscopy. Subsequently, the thermal stability, mechanical properties, and electrochemical performance of the films are evaluated. This systematic approach enables the correlation of structural and morphological features with the electrical behavior of the materials, providing a comprehensive understanding of their potential application in advanced electrochemical systems.

**Figure 2:**
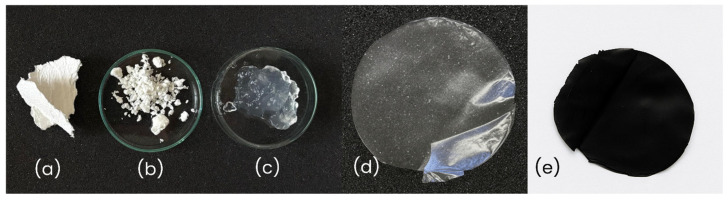
Raw materials and intermediate forms used in the preparation of films, (**a**) Sheet fragment of bleached kraft pulp (BKP), (**b**) Pelletized kraft pulp, and (**c**) Colloidal dispersion of SCNF, (**d**) Pure SCNF film, (**e**) rGO:SCNF film (1:5).

Figure 2 shows the morphological features observed at different stages during the sulfation process and film formation. Images (a–c) display the initial kraft pulp, its pelletized form, and the colloidal SCNF dispersion, while images (d–e) compare the films obtained from SCNF and SCNF:rGO, respectively, highlighting differences in color, homogeneity, and macroscopic appearance.

Based on the digital photographs taken against a neutral background, the visual appearance and color of the films were compared. Figure 2a,b show the characteristic features of cellulose, while Figure 2c presents the whitish transparent gel formed after obtaining SCNF. Figure 2d shows the SCNF film, which exhibits a translucent whitish tone typical of pure sulfated nanocellulose. In contrast, Figure 2e illustrates the change caused by the incorporation of rGO, which imparts a uniform opaque black color to the film, indicating its proper dispersion within the SCNF matrix. Both films maintain a continuous and flexible structure without visible cracks, confirming the successful formation of the hybrid film and its macroscopic homogeneity.

**Figure 3:**
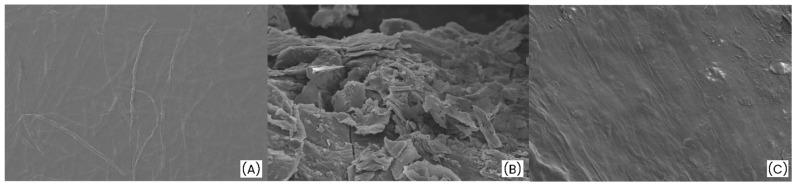
SEM micrographs of the analyzed samples: (**A**) SCNF film at 95×, (**B**) Reduce graphene oxide (rGO) at 4.00 k×, and (**C**) Surface of the SCNF:rGO hybrid film at 1.5 k×.

Scanning electron microscopy (SEM) was used to analyze the surface morphology of the obtained films. Figure 3A shows the micrograph corresponding to the sulfated cellulose nanofiber (SCNF) film at 95× magnification, where an interconnected network of continuous, entangled fibers forming a homogeneous and compact structure can be observed. This morphology reflects a successful sulfation process, resulting in a uniform and crack-free film. Figure 3B displays the morphology of reduced graphene oxide (rGO) at 4.00 k× magnification, characterized by an irregular texture and a layered structure, typical of exfoliated graphene sheets. Finally, Figure 3C presents the micrograph of the SCNF:rGO hybrid film at 1.5 k× magnification, where a more compact and continuous surface is observed compared to Figure 3A. This morphology suggests strong interfacial adhesion between the nanocellulose fibers and the rGO sheets. The observed morphological homogeneity supports the mechanical integrity of the material and will promote efficient electron transport.

**Figure 4:**
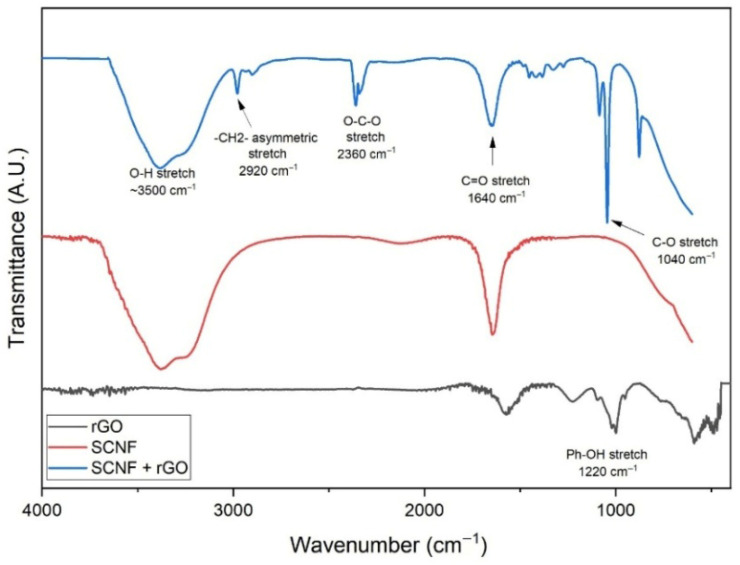
FTIR spectra of rGO (black), SCNF (red), and the SCNF:rGO hybrid film (blue).

The presence of functional groups in the samples was determined by analyzing the vibrational spectra obtained from FTIR. Figure 4 shows the spectra corresponding to SCNF, rGO, and the SCNF:rGO hybrid film. In the SCNF spectrum, the broad band observed around ~3500 cm^−1^ corresponds to O–H stretching vibrations of hydroxyl groups, while the signal at 2920 cm^−1^ is associated with asymmetric –CH_2_ stretching. Additionally, the band at 1640 cm^−1^ is related to carbonyl (C=O) stretching, and the signal at 1040 cm^−1^ confirms the C–O bond vibrations characteristic of sulfated cellulose. The rGO spectrum exhibits a signal at 1220 cm^−1^, attributed to the stretching of phenolic groups (Ph–OH), indicating the presence of a small amount of residual oxygenated groups after the GO reduction process. For the SCNF:rGO hybrid film, the characteristic bands of SCNF are preserved, although with changes in relative intensity, suggesting interactions between the hydroxyl and sulfate groups of nanocellulose and the remaining functional groups in rGO. These results confirm the integration of both components into a continuous hybrid network, supporting the good dispersion of rGO within the SCNF matrix.

**Figure 5:**
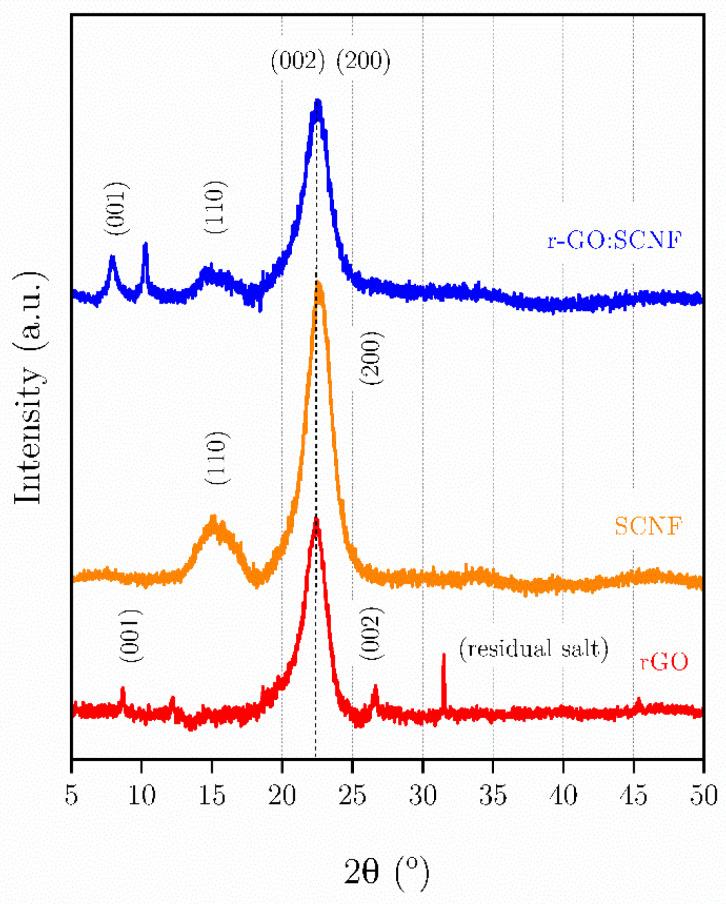
X-ray diffractograms of SCNF (orange), rGO (red) and the SCNF:rGO hybrid film (blue).

Figure 5 shows the X-ray diffraction (XRD) patterns of the SCNF, rGO, and SCNF:rGO (1:5) samples, and the crystallographic parameters extracted from each diffractogram are summarized in Table 2, including the diffraction angle (2θ), the Bragg angle (θ), and the corresponding interplanar spacing (d). The pure SCNF sample exhibits a characteristic peak at 2θ ≈ 22.59°, associated with the (200) reflection of cellulose type I, confirming the presence of crystalline domains within the nanofiber network. In contrast, the diffractogram of rGO shows a broad diffraction peak centered at 2θ ≈ 22.47°, corresponding to the (002) plane of reduced graphene oxide, which is indicative of its amorphous and turbostratic structure. For the hybrid SCNF:rGO (1:5) film, the main diffraction peak remains present but appears with lower intensity and slight broadening, shifting toward higher 2θ values (≈22.67°). Given the magnitude of this shift, which is close to the resolution limits of laboratory XRD measurements, a contribution from instrumental or data-processing uncertainty cannot be ruled out.

This behavior suggests a decrease in crystallinity and a reduction in interplanar spacing, from 3.9568 Å (rGO) to 3.9224 Å (SCNF:rGO). Although this shift toward higher 2θ values is subtle and lies close to the resolution limits of laboratory XRD measurements, it may be partly influenced by instrumental or data-processing uncertainties. Nevertheless, the observed displacement is consistent with trends reported in cellulose–graphene hybrid systems, where mild interfacial interactions—such as hydrogen bonding, electrostatic stabilization, or partial confinement of graphene sheets—can induce modest structural compaction without producing major rearrangements. Additionally, variations observed in the low-angle region (5–15°) are associated with rGO, confirming its effective incorporation into the nanocellulose network. Overall, these results demonstrate the successful integration of rGO within the SCNF matrix and the formation of a semicrystalline hybrid material in which both phases coexist. The slight peak broadening and the minor shift are therefore interpreted as suggestive—but not conclusive—evidence of limited SCNF–rGO interactions that generate a partially disordered and more compact structure, in agreement with the complementary morphological and electrochemical analyses.

**Figure 6:**
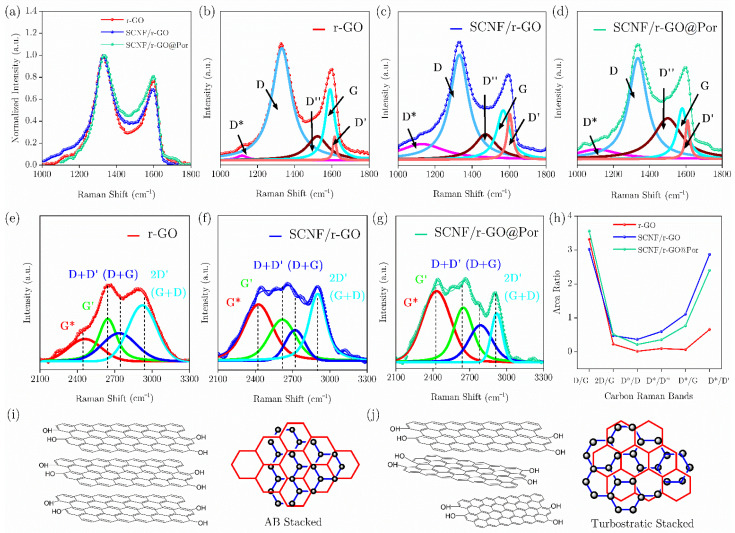
Raman spectra and structural analysis of SCNF:rGO and SCNF/rGO-porphyrin hybrid films. (**a**) Normalized Raman Spectra of r-GO, SCNF/r-GO and SCNF/r-GO@Por in the range 1000–1800 cm^−1^, (**b**) Deconvolution of the D and G bands for r-GO, showing the contributions of D*, D, D′ and G bands, (**c**) Deconvolution of Raman bands for SCNF/r-GO, highlighting the evolution of the defect-related bands, (**d**) Deconvolution of Raman bands for SCNF/r-GO@Por, indicating structural changes after porphyrin functionalization, (**e**) Second-order Raman spectrum of r-GO in the 2100–3300 cm^−1^ region with fitted contributions (D + D′, D + G, 2D and G + 2D), (**f**) Second-order Raman spectrum of SCNF/r-GO with corresponding deconvoluted bands, (**g**) Second-order Raman spectrum of SCNF/r-GO@Por and fitted peak components, (**h**) Area ratios of the main carbon Raman bands for each sample, (**i**) Schematic representation of AB stacked graphene layers and (**j**) Schematic representation of turbostratic stacked graphene structure.

The Raman spectra is presented in Figure 6. Both rGO and SCNF:rGO show the characteristic bands of reduced graphene: the D band at ~1340 cm^−1^, associated with defects and disorder, and the G band at ~1580 cm^−1^, corresponding to C=C bond vibrations in sp^2^ domains. In the SCNF:rGO hybrid sample, both bands exhibit higher relative intensity compared to pure rGO, reflected in an increased ID/IG ratio. This increase suggests a higher density of structural defects in rGO after incorporation into the sulfated nanocellulose matrix, consistent with literature reports of disorder induced by interactions with polymers [20]. Additionally, the elevated baseline at low wavenumbers is attributed to contributions from the SCNF matrix, confirming the effective integration of both components in the hybrid system.

Figure 6a shows the normalized Raman spectra of each sample: rGO, the sample modified with SCNF, and the cobalt porphyrin-functionalized sample (Por), in the spectral range of 1000–1800 cm^−1^.

The D band, located between 1330 and 1350 cm^−1^, arises from defects and disorder in the carbon lattice and from double-resonance processes near the K point at the edge of the Brillouin zone (BZ). The G band, centered at 1583 cm^−1^, corresponds to the Raman-allowed E_2_g optical phonon mode. The D″ band, between 1500 and 1550 cm^−1^, is associated with the amorphous phase, and its intensity is inversely proportional to the degree of crystallinity. The D′ band, around 1620 cm^−1^, corresponds to an intra-valley resonance with the G band and may exhibit splitting due to impurities. Finally, the D* band, in the range of 1050–1200 cm^−1^, originates from sp^3^ orbitals, like that observed in small nanocrystalline diamonds, sp^3^-rich phases, hexagonal diamonds, disordered graphite networks, polyenes, and trans-polyacetylene at grain boundaries.

The comparative Raman analysis of the three samples reveals that the incorporation of SCNF and subsequent functionalization with porphyrin induce significant structural and electronic modifications in rGO. The rGO sample exhibits a high ID/IG ratio of approximately 3.3 (Figure 6h), consistent with a high defect density and small graphitic domains. Such an outcome is expected for a partially reduced graphene oxide obtained by modified Hummers’ oxidation of graphite, followed by reduction, where disordered and residual oxygen-functionalized regions separate small sp^2^ fragments. The relatively large bandwidth (FWHM) of the D peak and the broadening of the G peak (FWHM ≈ 60 cm^−1^) indicate that the sample retains a considerable fraction of sp^2^ domains, albeit with a high degree of structural disorder (edges, vacancies, residual oxygenated groups, or small graphite crystal sizes). The moderate relative area of the D″ band (~14%) suggests the presence of locally distributed amorphous or sp^3^-character phases.

The addition of SCNF causes a notable increase in the D* contribution and a shift of the G peak to lower energies, indicating the appearance of new non-graphenic signals, likely associated with cellulose, due to overlapping vibrational modes of C–O and C–O–C groups and/or a higher fraction of sp^3^ at the interface. This may also be related to a modification of the electronic environment (strain or charge transfer) induced by interaction with SCNF. The slight relative decrease in the D intensity suggests spectral redistribution and/or partial separation of the sheets (intercalation dispersion), which reduces the fraction of edges or domains detected strictly as D. SCNF, due to its fibrillar morphology and functional groups (–OH, –SO_4_^−^), can adsorb onto the rGO sheets and prevent their restacking, keeping the sheets more separated and reducing edge-to-edge contact. Additionally, a mild reducing effect is possible. Hydroxyl groups, under certain conditions (temperature, acidic or basic pH, presence of catalysts), can act as a mild reducing agent. Together with the acidic nature of the sulfate group, this could modify the chemical microenvironment (local pH, protonation capacity) and promote dehydration or esterification reactions during thermal treatments, leading to oxygen loss from the sheets (partial reduction). Although the ID/IG ratio remains high (≈3.0), its decrease relative to rGO supports the hypothesis of the dispersing and reducing effect of nanocellulose.

The functionalization with porphyrin on the sample previously modified with SCNF induces clear Raman changes, consistent with significant structural and electronic perturbations. The I(D)/I(G) ratio increases to ≈3.55, higher than that of rGO and SCNF/rGO, along with a pronounced increase in the D″ band, which reaches 27.8% of the relative area and shows notable FWHM broadening. A decrease in D* is observed compared to the SCNF-modified sample (though still higher than in rGO), while the G peak occupies an intermediate position between the two. These results suggest an increase in local disorder and sp^3^/amorphous fraction in the carbon network, attributable to (i) a possible strong functionalization of the surface and (ii) the formation of chemical defects during porphyrin attachment. The partial shift of the G peak also indicates an electronic effect of the porphyrin (partial doping, strain compensation, or charge transfer) distinct from that induced by SCNF alone.

Figure 6e–g shows the second-order spectra (2100–3300 cm^−1^), where the combined bands D + D′, G′ (2D), G*, and 2D′ are observed, corresponding to the double-resonance modes characteristic of graphene and its derivatives. In rGO (Figure 6e), the 2D band is broad and poorly defined, indicative of turbostatic stacking. In the SCNF/rGO sample (Figure 6f), the 2D band intensifies and shifts slightly, suggesting partial reordering of the sheets and possible π–π interactions between SCNF and rGO, which reduce interlayer disorder. Finally, in the SCNF/rGO/porphyrin sample (Figure 6g), the 2D band broadens again, accompanied by a more defined appearance of the combined bands (D + D′ and G + D), evidencing a synergistic interaction between the polymeric matrix (SCNF), the graphene layers, and the porphyrin, resulting in a hybrid structure with modified electronic characteristics.

Overall, the Raman results confirm the effective interaction between sulfated cellulose nanofibers, reduced graphene oxide, and porphyrin, as evidenced by variations in the D, G, and 2D bands, peak shifts, modulation of intensity ratios, and changes in stacking modes. These observations support the formation of a SCNF/rGO-porphyrin hybrid network with π–π interactions and/or partial covalent bonds between the phases, consistent with the expected structural behavior of functionalized composite materials.

**Figure 7:**
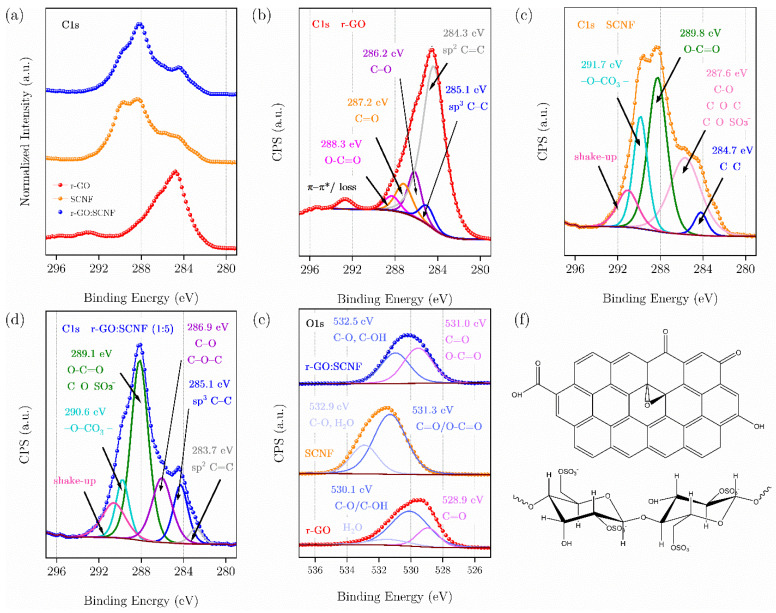
High-resolution XPS analysis of the carbon (C 1s) and oxygen (O 1s) core levels. (**a**) Normalized intensity of the three samples. (**b**–**d**) reduced graphene oxide r-GO (red), sulfated cellulose nanofibers SCNF (orange), rGO:SCNF composite mass ratio 1:5 (blue). (**e**) comparative O 1s envelopes. (**f**) schematic representations of the representative structures r-GO and sulfated cellulose.

High-resolution XPS provides direct evidence that both the graphene and the nanocellulose phase in the hybrid composite are not merely physically mixed, but also chemically and electronically coupled through surface functional groups. The individual C 1s and O 1s envelopes of r-GO and SCNF (Figure 7b,c) define two distinct chemistries, and the rGO:SCNF composite (Figure 7d) inherits features from both, demonstrating the strong interfacial integration. The corresponding survey spectra, shown in Figure S3, from Supporting Information corroborate these assignments at the elemental level: pristine r-GO shows only C and O, whereas SCNF exhibits intense O, a clear S 2p signal associated with sulfate in S^6+^ oxidation state, and a detectable N 1s component attributed to urea-derived carbamate/amide-like fragments. The composite spectrum contains all these signals simultaneously (C, O, S, N), unambiguously confirming that the sulfated nanocellulose and r-GO are co-present in a single surface environment.

For rGO (Figure 7b), the C 1s envelope is dominated by the sp^2^ C=C component (≈284.3 eV), which we use as an internal reference for binding-energy alignment because it represents the graphitic domains. A second contribution at ≈285.1 eV corresponds to sp^3^ C–C/C–H and disordered edge carbon. Progressively higher binding-energy components reflect increasing oxygen content and oxidation state: C–O/C–OH/epoxy/ether species appear at ≈286.2 eV; carbonyl/quinone/lactone-like C=O at ≈287.2 eV; and carboxylate/O–C=O moieties at ≈288.3 eV. Finally, a broad loss/π–π* feature emerges at high binding energy (≈290–292 eV equivalent shift relative to the main sp^2^ peak), which is characteristic of inelastic shake-up processes in conjugated sp^2^ systems and is widely reported in reduced graphene oxide. Such spectral peaks, taken together, potentially demonstrate that most of the lattice exhibits an sp^2^ character, while residual epoxide, hydroxyl, and carboxylate sites remain at basal-plane defects and sheet edges.

The C 1s spectrum of the sulfated cellulose nanofibers (SCNF, Figure 7c) is qualitatively different. The lowest-binding-energy contribution near 284.7 eV is attributed to aliphatic C–C/C–H environments. The bulk of the envelope, however, is shifted to higher binding energy, reflecting the highly oxygenated and partially ionized nature of the sulfated polysaccharide. A strong component at ≈287.6 eV arises from C–O/C–O–C linkages intrinsic to cellulose (glycosidic ether bridges and pendant hydroxyls). A closely related component, assigned here to C–O–SO_3_^−^ (sulfate ester) introduced by thermal sulfation with sulfamic acid and urea, also falls in this range but tends to extend the tail of the envelope to higher binding energy due to the strongly electron-withdrawing S^6+^ center. At still higher binding energy, a distinct contribution at ≈289.8 eV is assigned to O–C=O/carboxylate-like or carboxylated termini, consistent with oxidative cleavage/partial carboxylation of the nanofiber surface under the sulfation conditions. We also observe a weak feature near ≈291–292 eV, which we attribute primarily to chemically bound carbonate-/bicarbonate-like O–C=O species stabilized by the highly polar, sulfate- and carboxylate-rich SCNF surface, although a minor contribution from residual CO_2_-derived contamination cannot be completely ruled out. This pronounced high-BE tail, absent in pristine r-GO, indicates that the SCNF surface is not merely hydroxylated (cellulose-like) but bears highly charged, strongly bound oxyanion functionality. The presence of S in the SCNF survey spectrum (Figure S3) and the expected S 2p signal at binding energies consistent with sulfate (S^6+^) directly support this assignment.

The O 1s region (Figure 7e) reinforces this picture. For rGO, O 1s can be decomposed into (i) a lower-binding-energy contribution associated with carbonyl/carboxylate-type oxygen (C=O/O–C=O, i.e., quinone-like and edge carboxylates), and (ii) a higher-binding-energy contribution attributed to C–O/C–OH/epoxy/ether groups and strongly hydrogen-bonded surface water. This is entirely consistent with partially reduced graphene oxide, where residual epoxy/hydroxyl functionalities coexist with oxidized edge sites.

In contrast, SCNF displays an O 1s envelope in which the dominant high-binding-energy component is broader and shifted to even higher binding energy than in r-GO. We assign this dominant component to oxygen in C–O/C–OH/C–O–C environments of cellulose, but crucially also to the sulfate ester oxygen (–OSO_3_^−^) created by sulfamic-acid/urea treatment. The sulfated cellulose surface is strongly anionic and highly hydrated; therefore, this contribution also captures tightly bound, hydrogen-bonded water and partially protonated sulfate species. A lower-binding-energy shoulder in SCNF O 1s corresponds to more “carbonyl-like” oxygen (C=O/O–C=O), i.e., oxidized termini, carboxylate/carboxylated sites, and possible carbamate/amide-type N–C=O fragments resulting from reaction with urea at 150 °C. The appearance of N 1s in the SCNF survey (Figure S2) supports the presence of these nitrogen-containing functionalities.

The rGO:SCNF composite (Figure 7d for C 1s, and Figure 7e for O 1s, blue traces) contains all these signatures simultaneously. In C 1s, the composite retains a clear sp^2^ C=C component at low binding energy (graphitic r-GO domains), but also exhibits the high-binding-energy components characteristic of SCNF: C–O–SO_3_^−^/polysaccharide C–O–C, O–C=O/carboxylate near 289–290 eV, and the carbonate-like shoulder near 290–291 eV. In other words, the carbon spectrum of the composite cannot be described as a simple superposition of “graphite-like” r-GO plus “neutral cellulose.” Instead, it reflects a chemically integrated interface in which r-GO sheets coexist with (and presumably anchor onto) a highly oxidized, sulfated, partially carboxylated polysaccharide surface.

Likewise, the O 1s envelope of the composite shows both families of oxygen: a “carbonyl/carboxylate-like” component comparable to that in r-GO (C=O/O–C=O at lower BE) and a “sulfated cellulose-like” high-BE component associated with sulfate ester oxygen, cellulose C–O/C–OH, and strongly hydrogen-bonded interfacial water. The relative areas of these two O 1s contributions in the composite are comparable, indicating that neither phase is spectroscopically silent at the surface (See Table S1 from Supporting Information). This directly implies that r-GO is not fully encapsulated on the SCNF surface, nor is SCNF completely masking r-GO; rather, both are exposed to the XPS probe volume, signaling intimate but co-accessible interfacial contact. Such a result strongly suggests that the interface between rGO and SCNF is not purely van der Waals; instead, it likely could involve hydrogen bonding, electrostatic interactions between the anionic sulfate/carboxylate sites on SCNF and the residual oxygenated/defective sites on rGO, and possibly carbonate/carbamate bridging.

In summary, XPS demonstrates that sulfation (and mild oxidative functionalization) of cellulose nanofibers, as confirmed by the S 2p and N 1s signals in the survey spectra (Figure S3), creates a highly anionic polysaccharide surface. When mixed with rGO, this surface does not passivate the graphene sheets but instead forms an integrated hybrid in which both phases remain spectroscopically active at the outer surface. This interfacial chemistry is expected to underline the improved colloidal stability, interfacial charge transfer, and mechanical coupling observed for the SCNF:rGO composite.

To further assess the structural consistency among samples, the crystallinity index (CrI) was calculated for multiple independently prepared films using the Segal method applied to the XRD diffractograms. The CrI values showed only minor variations across batches, indicating that the DES-mediated sulfation process and subsequent hybrid formation do not introduce significant crystallographic heterogeneity. Similarly, Raman spectra collected at several randomly selected points on each film yielded highly reproducible ID/IG ratios, with variations remaining within ±0.1–0.2 units. No anomalous regions or abrupt changes were detected, suggesting that rGO is uniformly dispersed and retains comparable defect density throughout the films. These observations, together, confirm that the structural features of the SCNF:rGO hybrids remain consistent across independently prepared samples.

**Figure 8:**
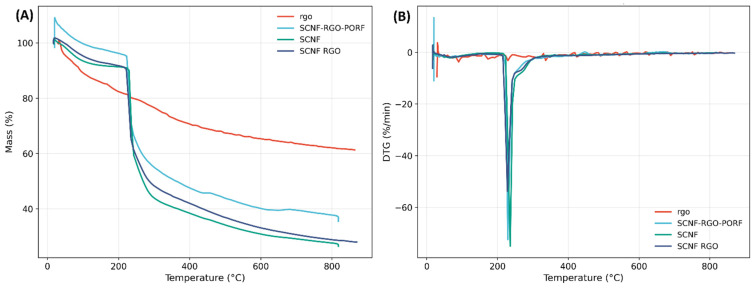
(**A**) Thermogravimetric (TGA) curves and (**B**) derivative (DTG) curves for rGO, SCNF, the SCNF:rGO and SCNF:rGO: PORF hybrid film.

Figure 8A,B show the thermal degradation profiles obtained by thermogravimetric analysis (TGA) and derivative thermogravimetry (DTG) for the rGO, SCNF, SCNF:rGO (1:5), and SCNF-rGO-PORF samples. The characteristic parameters, including onset degradation temperature, maximum degradation temperature, and residual mass at 800 °C, are summarized in Table 3.

The samples exhibit a multistage thermal degradation profile, characteristic of cellulose-based materials containing carbonaceous phases. The TGA curves show an initial mass loss between 30 °C and 120 °C, attributed to the removal of moisture and adsorbed molecules, followed by a major degradation stage between 200 °C and 350 °C, corresponding to the thermal decomposition of the cellulose structure, associated with the cleavage of glycosidic bonds and chain depolymerization. In the same zone Nurazzi et al. (2021) reported in their review of TGA behavior in cellulose fibers that the primary mass-loss stage (≈200–350 °C) is related to dehydration, bond scission, and the release of light volatile species, driven by the evolution of oxygen-containing molecules and chain fragments [21], which is coincident with our findings. For pure SCNF, a single, sharp DTG peak is observed around 235 °C, confirming a homogeneous decomposition event associated with the cleavage of glycosidic bonds. In contrast, the SCNF:rGO (1:5) hybrid film shows a broader DTG signal with a slight shift to lower temperatures (≈228 °C), indicating partial stabilization of the cellulose phase due to the interaction with rGO sheets, which favors gradual heat transfer and delays chain scission. The rGO:SCNF–PORF film exhibits a less intense and more displaced DTG minimum, suggesting that the presence of porphyrin introduces additional crosslinking or π–π interactions that improve thermal resistance. These observations demonstrate that the incorporation of rGO and porphyrin modifies the degradation pathway, resulting in a more thermally stable and structurally reinforced hybrid network.

According to the results presented in Table 3, the rGO sample exhibited the lowest initial mass loss among the analyzed materials, yet showed the highest final residue, which is consistent with its predominantly carbonaceous composition. This behavior can be attributed to the removal of most oxygen-containing functional groups during rGO synthesis. Under an inert atmosphere, the initial mass loss mainly corresponds to the desorption of moisture, residual solvents, or volatile functional groups, while the remaining sp^2^-carbon network remains stable up to significantly higher temperatures. Supporting this observation, an interlaboratory study on the TGA behavior of graphene-based materials reported that high residue values are typical of carbon-rich systems with low oxygen functionality [22].

In contrast, the pure SCNF sample exhibits a pronounced degradation event centered at approximately 235 °C and a low final residue (27.6%), behavior characteristic of sulfated nanocellulose, which shows reduced thermal stability due to the presence of –SO_3_H groups introduced during sulfation. This phenomenon is consistent with SCNF prepared via sulfamic acid treatment, since the incorporation of sulfate groups generates thermally labile sites within the cellulose structure. It has been reported that sulfated cellulose produced via DES exhibits decreased thermal stability due to the presence of sulfate ester linkages (C–O–S), which promote earlier polysaccharide chain degradation [22].

The onset degradation temperature of the SCNF:rGO hybrid (≈105 °C) appears lower than that of pristine SCNF (≈80 °C); this trend is expected due to the presence of –SO_3_^−^ groups introduced during DES-based sulfation, which promote early acid-catalyzed dehydration. In contrast, rGO contributes to stability at intermediate and high temperatures through barrier effects, improved heat dissipation, and greater char formation, leading to higher Tmax and a larger residual mass at 800 °C. Thus, the lower onset temperature reflects the intrinsic behavior of sulfated cellulose, while the enhanced stability at higher temperatures arises from rGO, with both effects acting complementarily in the hybrid.

Although the thermal analysis suggests a slight improvement in stability with the addition of rGO and porphyrin, the associated mechanical reinforcement could not be quantitatively demonstrated. Preliminary tensile and flexibility tests were attempted; however, the results were not sufficiently consistent to support a conclusive analysis, largely due to the sensitivity of the films to drying and handling conditions. For this reason, the quantitative data was omitted. Even so, the hybrid films consistently exhibited better cohesion and lower fracture tendency during practical manipulation, supporting the qualitative notion of improved robustness.

Overall, these results demonstrate a synergistic interaction between the organic and carbonaceous phases that enhances the thermal resistance of hybrid films, supporting their potential use as functional materials for electrochemical or energy-related applications.

### Electrochemical Analysis

Based on the results obtained from the initial characterizations (macroscopic, microscopic, and thermal), pure SCNF was selected as the reference system for subsequent electrochemical tests. Building on this, rGO was incorporated to evaluate the variability of electrochemical properties.

To assess the behavior of electrodes prepared with the films, the electrochemical response of the Fe (CN)_6_^3−^/^4−^ redox pair was studied to determine the real surface areas (Figure S1), as well as the oxidation of hydrazine. Figure 8A shows the cyclic voltammograms (CV) of the GC electrode and SCNF-rGO electrode in a 5 mM hydrazine solution. The oxidation of hydrazine occurs in the same potential region for both electrodes; however, the electrode with the SCNF-rGO film exhibits a slight electrocatalytic effect, with the anodic peak potential decreasing from 0.7 V to 0.66 V.

**Figure 9:**
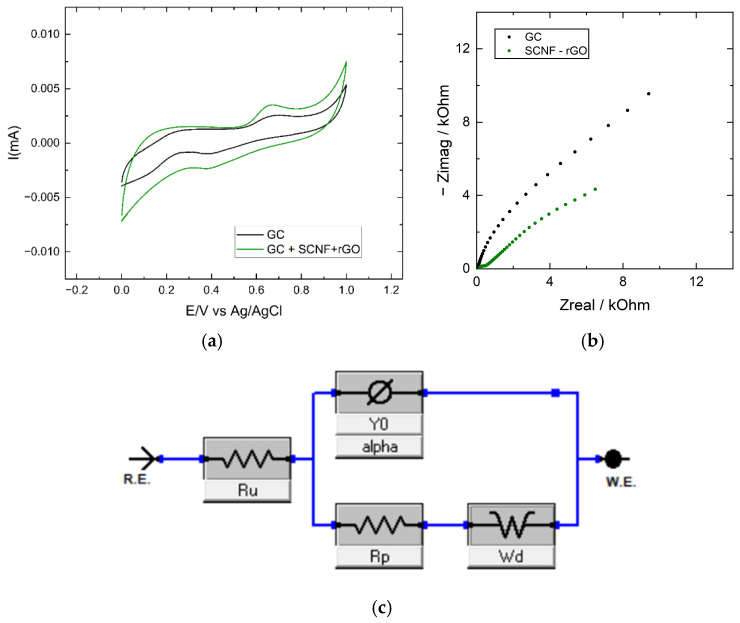
(**a**) Cyclic voltammograms of GC (black) and SCNF-rGO (green) in 5 mM hydrazine solution in PBS, pH 7.04, at 100 mV s^−1^. (**b**) Nyquist plots recorded in 0.1 M K_3_Fe (CN)_6_/1 M KNO_3_ at the equilibrium potential with a 10 mV AC perturbation for GC (black) and SCNF-rGO (green). (**c**) Experimental design of the equivalent circuit used for fitting.

The main difference between the electrodes lies in the capacitive currents: the SCNF-rGO film electrode exhibits higher current values, which can be attributed to its larger effective surface area and, consequently, an increase in capacitive contributions. The active surface areas calculated from the data in Figure S1 are 0.03 cm^2^ for the GC electrode and 0.093 cm^2^ for the SCNF-rGO electrode. To further understand this behavior, electrochemical impedance spectroscopy (EIS) was performed, as shown in Figure 9b.

Figure 9b presents the Nyquist plots obtained for the bare GC and SCNF:rGO (1:5) electrodes. Both systems exhibit the characteristic features of an electrochemical interface consisting of a semicircle in the high-frequency region followed by a straight line in the low-frequency region.

The SCNF:rGO electrode displays a smaller semicircle diameter compared to the GC electrode, indicating a lower charge-transfer resistance (Rct) and a faster electron-transfer process. Additionally, at low frequencies, the plot of the SCNF: rGO electrode shows a more pronounced 45° slope, confirming that ionic diffusion dominates the impedance response in this region. This behavior suggests that the hybrid film facilitates both electron and ion transport due to its porous and conductive structure.

From Figure 9b, the data were fitted using a Randles circuit including a Warburg element to account for diffusion.

The impedance data of all electrodes were fitted using a classical Randles-type equivalent circuit (Rs–(CPE‖Rct)–W), which is the standard and most physically appropriate model for the Fe(CN)_6_^3−^/^4−^ redox couple. This system exhibits semi-infinite linear diffusion and well-defined electron-transfer kinetics, and its interfacial behavior is accurately represented by the CPE–W Randles configuration.

To evaluate the quality of the fits, and reduced chi-square (${\chi^{2}}_{red}$) values were calculated from the residuals (Residual Zreal and Residual Zimag) obtained through Gamry Echem Analyst. The GC electrode exhibited $\chi^{2}$ = 0.6735 and ${\chi^{2}}_{red}$ = 0.0120, indicating an excellent agreement between the experimental and simulat ed impedance spectra. Similarly, the SCNF:rGO film showed $\chi^{2}$ = 2.0532 and ${\chi^{2}}_{red}$ = 0.0367, confirming that the Randles CPE–W circuit provides an accurate and physically meaningful description of the hybrid interface. In all cases, the residuals displayed a random distribution without systematic deviations, further supporting the suitability of the selected model.

The values of the circuit elements for each spectrum are summarized in Table 4. The ohmic resistance increases from 46 Ω for GC to 140 Ω, reflecting the intrinsic resistance of the SCNF-rGO film. The capacitance was modeled using a constant phase element (CPE), where the exponent α decreases from a value close to 1 for GC to 0.8 for the film. This reduction reflects the increased surface irregularity of the film, consistent with the morphological features observed in the SEM images of Figure 3.

**Figure 10:**
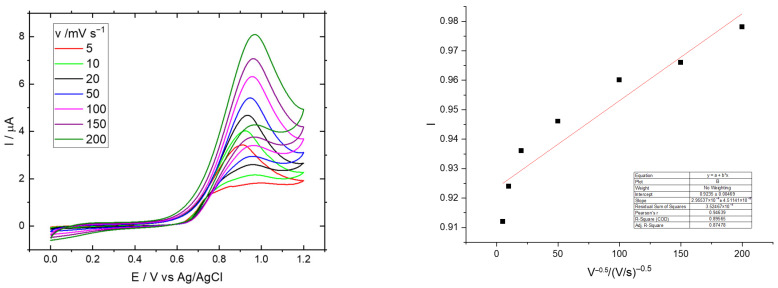
Cyclic voltammograms of the glassy carbon (GC) electrode in 5.0 mM hydrazine solution at different scan rates (5–200 mV s^−1^), showing the increase in anodic current with scan rate.

The SCNF:rGO film demonstrated suitable electrochemical properties for functioning as an electrode, and the hydrazine oxidation exhibited higher peak currents compared to the GC electrode (Figure 10). This figure shows a series of CV curves (I vs. E) recorded at different scan rates (5 to 200 mV s^−1^). In all measurements, a single anodic peak is observed, with the peak current increasing as the scan rate increases. The progressive increase in peak current (Iₚ) with scan rate (v) indicates that the electrochemical process is diffusion-controlled, with redox species moving toward the electrode surface. The sharp and well-defined peak morphology supports quasi-reversible behavior, without evidence of coupled chemical reactions, as no additional distortions are observed in the curves. The linearity of the plot confirms that the peak current (Iₚ) is directly proportional to the square root of the scan rate (v^1/2^), which is characteristic of a diffusion-controlled process rather than surface adsorption. The high linear correlation coefficient (R^2^ ≈ 0.98) demonstrates excellent adjustment of the measurements to the model.

**Figure 11:**
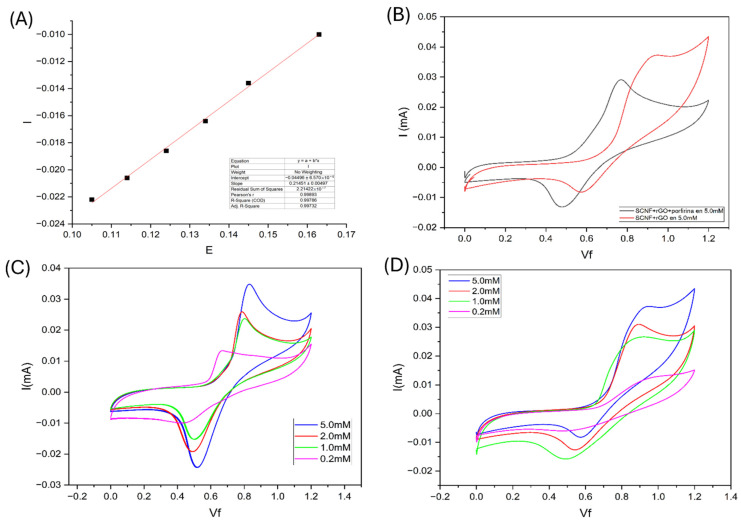
Cyclic voltammograms recorded for SCNF: rGO and SCNF-rGO-Porphyrin films in hydrazine solution: (**A**) calibration curves of anodic peak current versus concentration, used to determine the sensitivity of each electrode, (**B**) comparison at 5 mM, and (**C**,**D**) effect of hydrazine concentration (0.2–5.0 mM).

The electrode was modified with an electrocatalytic molecule, octaethylporphyrin (OEP), a component widely reported as an effective electrocatalyst for hydrazine electro-oxidation [19]. Figure 11A shows a comparison of the voltammograms obtained in the presence of 5 mM hydrazine for the SCNF:rGO and SCNF:rGO:POR electrodes. The oxidation peak shifts to lower potential due to the presence of porphyrin on the electrode surface, indicating a reduction in the activation energy of the process. This result confirms that the SCNF:rGO film serves as a suitable platform for immobilizing electrocatalysts and that its modification enhances the kinetics of hydrazine oxidation.

Figure 11B,C show the effect of hydrazine concentration (0.2–5.0 mM) on the voltametric response of both electrodes, while Figure 11D presents the calibration curves of anodic peak current (Iₚ) versus concentration, used to determine the sensitivity of each system. From these curves, slopes of 0.32187 µA·mM^−1^ for SCNF:rGO and 0.21451 µA·mM^−1^ for SCNF:rGO-Por were obtained, indicating that both systems exhibit comparable sensitivities and are suitable for application in electrochemical sensors for hydrazine detection.

Using the calibration slope obtained for SCNF:rGO–Por (0.21451 µA·mM^−1^), the analytical performance of the system was further evaluated by estimating the limit of detection (LOD) and limit of quantification (LOQ). The LOD and LOQ were calculated using the 3σ/m and 10σ/m criteria, respectively, where σ corresponds to the standard deviation of the blank current (film in electrolyte without hydrazine). The resulting values were LOD = 1.6 × 10^−5^ M (≈16 µM) and LOQ = 5.4 × 10^−5^ M (≈54 µM). Although these values are higher than those typically reported for highly optimized rGO- or metalloporphyrin-based hydrazine sensors—which can achieve sub-micromolar or even nanomolar LODs when pulse techniques such as DPV or amperometry are employed—it is important to note that the objective of this work was not to minimize the detection limit. Instead, our aim was to demonstrate the feasibility, robustness, and operational stability of a sustainable SCNF:rGO–Por hybrid platform operating under neutral pH conditions and evaluated through simple cyclic voltammetry.

The long-term operational stability of the modified electrodes was evaluated by cyclic voltammetry over 10 consecutive cycles in hydrazine solutions. The SCNF:rGO–Por electrode showed highly reproducible voltammetric profiles throughout the cycling sequence, with no measurable decrease in peak current and no significant shifts in oxidation potential. These results confirm that the hybrid system maintains its electrochemical activity under repeated redox cycling.

Although the decrease in oxidation potential achieved by the SCNF:rGO–Por electrode (≈40 mV) may appear modest, this shift is nevertheless relevant for practical electrochemical applications. Even small reductions in overpotential are advantageous under real sensing conditions, as they decrease the likelihood of interference from species that oxidize at higher potentials and help reduce background current. Such shifts often reflect improved charge-transfer kinetics, which are consistent with the lower Rct, and higher peak currents observed for the hybrid electrodes. In applied systems such as hydrazine sensors or anodic layers in direct hydrazine fuel cells, even small decreases in overpotential can contribute to faster response times, improved signal-to-noise ratio, and reduced energy losses.

The electrochemical analyses confirm that the SCNF:rGO hybrid films exhibit a diffusion-controlled response. This behavior is consistent with that reported for GC electrodes, where hydrazine oxidation occurs under diffusion control and charge transfer remains quasi-reversible. Moreover, the slight decrease in the overpotential oxidation observed for the SCNF:rGO electrode compared to GC indicates more favorable oxidation kinetics, consistent with the presence of go sheets that facilitate electron transfer and enhance the system’s conductivity. The incorporation of rGO into the SCNF matrix produces two main effects: (i) an increase in capacitive current, attributable to the larger electroactive surface area generated by the porous film structure, and (ii) a moderate reduction in oxidation potential, consistent with improved electron-transfer kinetics. These results agree with previous studies on graphene-based composites, such as rGO/PEDOT: PSS or GO/conductive polymers, which report similar behavior: higher peak currents, lower overpotentials, and improved response toward redox analytes [23,24,25].

Compared to the biopolymer/graphene or rGO–polymer electrodes described above, the SCNF:rGO platform offers additional practical advantages derived from its highly loaded, hydrophilic, and binder-free architecture. The sulfated nanocellulose matrix ensures excellent dispersion of rGO without surfactants or polymeric binders, preventing graphene rearrangement and preserving continuous conductive pathways. Furthermore, metal-free DES synthesis with minimal solvents produces a clean interface, free of residual reducing agents or catalysts, improving durability and electrochemical stability. The mechanically robust and flexible SCNF network forms a self-supporting film, eliminating the need for inert polymer matrices such as PVDF or Nafion, and the anionic sulfate groups improve ionic mobility and charge transfer efficiency. Together, these characteristics position the SCNF:rGO hybrid as a sustainable, high-performance electrode with superior conductivity, improved ion transport, and greater compatibility with catalytic molecules compared to traditional biopolymer/graphene systems.

Also, during experimentation, the superior performance of the 1:5 (rGO:SCNF) ratio can be rationalized by considering the balance between percolation of the conductive phase and structural reinforcement provided by the nanocellulose matrix. At lower rGO loadings, the graphene domains do not form a continuous conductive network, limiting electron transport; whereas higher loadings (e.g., 2:3) disrupt the hydrogen-bonded SCNF framework and lead to mechanical fragility and poor film cohesion. The 1:5 composition lies near the percolation threshold at which rGO sheets establish effective electronic pathways while remaining stabilized by the highly charged, sulfated SCNF network. This synergistic balance explains why 1:5 yields homogeneous, flexible, and electrochemically efficient hybrid films.

Furthermore, the incorporation of octaethylporphyrin (OEP) into the SCNF:rGO hybrid film led to an additional shift of the oxidation peak toward lower potentials, indicating a reduction in the activation energy of the process and enhanced oxidation kinetics. This behavior is consistent with reports on rGO–porphyrin hybrid materials, where π–π interactions between graphene sheets and the aromatic porphyrin rings facilitate electron transport and promote the formation of more stable intermediate species. Previous studies, such as Dreyse Silva, have demonstrated that functionalization with a porphyrin macrocycle significantly improves electrocatalytic activity, in agreement with the findings of the present work [26]. Overall, our results are consistent with reports on cellulose–graphene hybrid systems, in which the combination of a biopolymeric matrix with carbonaceous phases leads to structural and functional synergy: cellulose provides flexibility, stability, and mechanical support, while rGO and porphyrin supply conductivity and active sites for charge transfer.

Despite the promising short-term electrochemical performance, the long-term behavior of SCNF:rGO films under practical operating conditions remains an open question. Future studies will address extended cycling stability, electrolyte-dependent durability, mechanical robustness under compression or hydration–dehydration cycles, and integration into functional device architectures such as fuel cells or commercial electrochemical sensors.

## 4. Conclusions

The structural, thermal, and electrochemical results confirm that the SCNF: rGO hybrid films possess a well-integrated structure, in which the sulfated cellulose nanofibers and reduced graphene oxide combine homogeneously, exhibiting good dispersion and strong interfacial interactions, as evidenced by SEM, FTIR, XRD, XPS, and Raman analyses. In contrast to most cellulose–graphene composites previously reported, which rely primarily on physical mixing and limited interfacial compatibility, the DES-sulfated nanocellulose used here provides a highly charged, chemically interactive matrix that promotes intimate dispersion of rGO sheets and enhances the structure–function relationship responsible for the improved electrochemical performance [27]. The incorporation of porphyrin modifies the electronic and structural properties, enhancing the catalytic response toward hydrazine oxidation. TGAs demonstrate increased thermal stability compared to pure SCNF, while electrochemical studies show higher peak currents, lower overpotential, and more efficient charge transfer. Overall, these results demonstrate that the developed materials are sustainable, stable, and electrochemically active, positioning the SCNF: rGO: POR films as promising candidates for application in electrochemical sensors for hydrazine and other redox-active analytes.

## Figures and Tables

**Table 1:** Composition of SCNF: rGO Hybrid Films.

SAMPLE	SCNF (g)	rGO (g)	2-Propanol (mL)	Notes
SCNF (pura)	12.5			Control
SCNF:rGO	12.5	0.03	10	Ratio (1:5) rGO:SCNF
SCNF:rGO	7.5	0.06	20	Ratio (2:3) rGO:SCNF

**Table 2:** Crystallographic parameters of SCNF, rGO, and SCNF:rGO (1:5) obtained from X-ray diffraction (XRD).

SAMPLE	2θ	θ	θ RAD	SEN (θ RAD)
SCNF	22.47	11.235	0.1961	0.1948
rGO	22.59	11.295	0.1971	0.1958
SCNF:rGO	22.67	11.335	0.1978	0.1965

**Table 3:** Characteristic thermal parameters obtained from TGA for the different film formulations.

SAMPLE	T on Set (°C)	T Max (°C)	Residual at 800 °C (%)
rGO	56.1	29.0	62.1
SCNF	80.1	235.9	27.6
rGO:SCNF 1:5	105.3	228.2	28.9
rGO:SCNF-POR	229.3	229.3	37.7

**Table 4:** Values of the respective elements in the Randles circuit used to fit the data from Figure 8B.

Electrode	Rs [Ω]	Q [S·s^α^]	Alpha [α]	R [Ω]	W [S·s^1/2^]
GC	46.82	15.28μ	0.962	2.723	76.8μ
SCNF:rGO	140.1	10.41μ	0.806	356.7	485.7μ

## Data Availability

The raw data supporting the conclusions of this article will be made available by the authors on request.

## Supplementary Materials

The following supporting information can be downloaded at: https://www.mdpi.com/article/10.3390/polym17233225/s1, Figure S1: Cyclic voltammograms at different scan rates; Figure S2: Nyquist plots and charge-transfer resistance analysis; Figure S3: XPS survey spectra of rGO, SCNF, and rGO:SCNF composite. Table S1: Presents the high-resolution XPS deconvolution of the C1s and O1s regions for r-GO, SCNF, and the r-GO/SCNF hybrid film.
