# Supplementary material for: From Cellulose to Functional Electrode SCNF:rGO Hybrid Films for Electrochemical Applications
Source: Polymers (Basel). 2025 Dec 4;17(23):3225. doi: 10.3390/polym17233225 (PMC12694374; doi:10.3390/polym17233225)
Supplement: Supplementary file 1 [file polymers-17-03225-s001.zip › polymers-3998094-supplementary.pdf]

From Cellulose to Functional Electrode SCNF: rGO Hybrid Films for Electrochemical Applications

Josefa Silva[1], J.R Sosa-Acosta[2,3], Galo Ramírez[2,3], Katherina Fernández[1,*], Rodrigo del Rio[2,*]

[11]Laboratorio de Biomateriales, Departamento de Ingeniería Química, Facultad de Ingeniería, Universidad de Concepción, Chile.

[2]Departamento de Química Inorgánica, Facultad de Química, Pontificia Universidad Católica de Chile, Av. Vicuña Mackenna 4860, Casilla 306, Correo 22, Santiago 8331150, Chile

[3]Millennium Institute on Green Ammonia as Energy Vector (MIGA), Av. Vicuña Mackenna 4860, Macul, Santiago 7820436, Chile

*Corresponding authors: kfernandeze@udec.cl; rdelrioq@uc.cl,

Figures

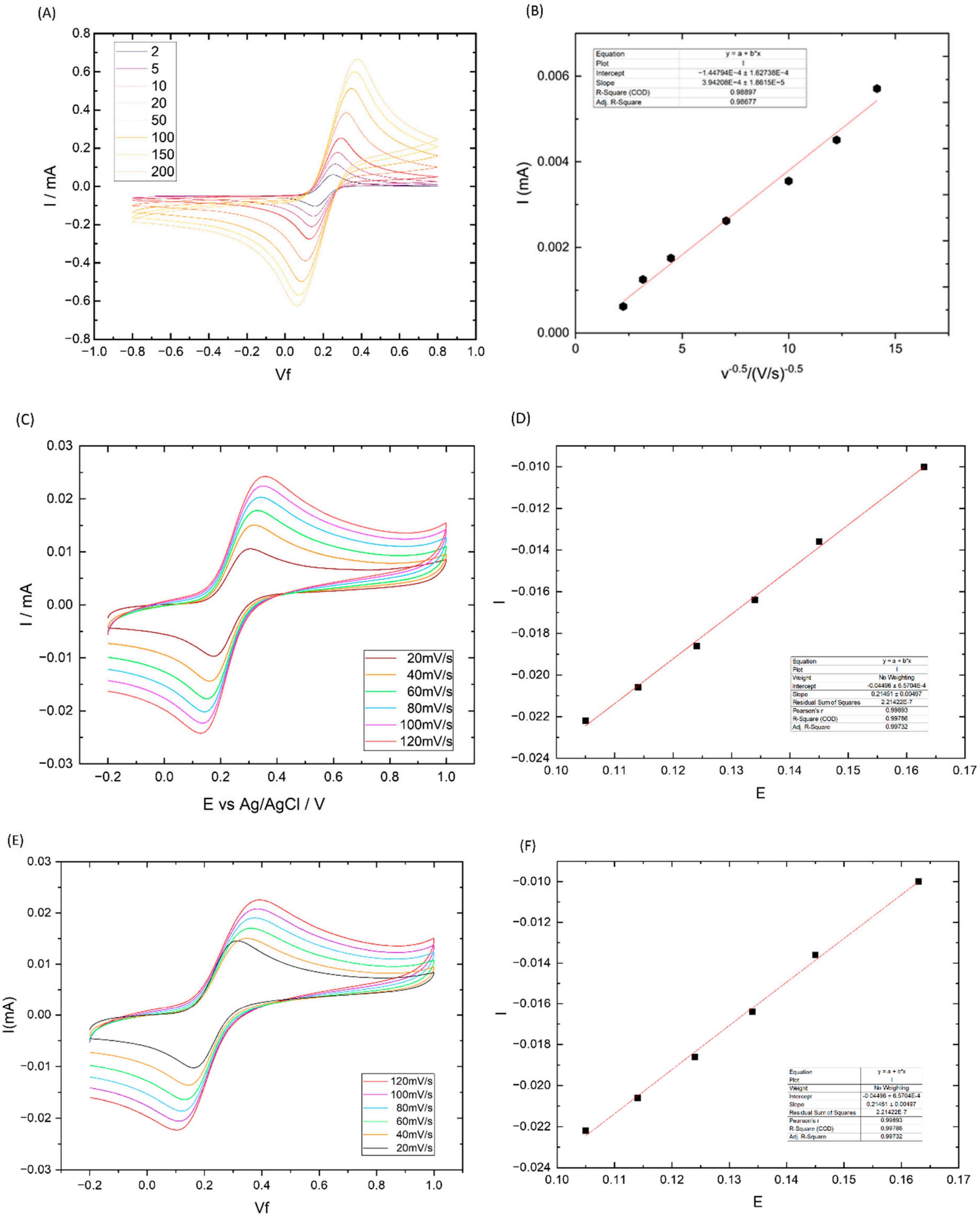
(A)
I / mA
Vf
2
5
10
20
50
100
150
200
(B)
Equation
y = a + b*x
Plot
I
Intercept
−1.44794E−4 ± 1.62738E−4
Slope
3.94208E−4 ± 1.8615E−5
R-Square (COD)
0.98897
Adj. R-Square
0.98677
I (mA)
v^-0.5/(V/s)^-0.5
(C)
I / mA
E vs Ag/AgCl / V
20mV/s
40mV/s
60mV/s
80mV/s
100mV/s
120mV/s
(D)
I
E
Equation
y = a + b*x
Plot
I
Weight
No Weighting
Intercept
-0.04496 ± 6.5704E-4
Slope
0.21451 ± 0.00497
Residual Sum of Squares
2.21422E-7
Pearson's r
0.99893
R-Square (COD)
0.99786
Adj. R-Square
0.99732
(E)
I(mA)
Vf
120mV/s
100mV/s
80mV/s
60mV/s
40mV/s
20mV/s
(F)
I
E
Equation
y = a + b*x
Plot
I
Weight
No Weighting
Intercept
-0.04496 ± 6.5704E-4
Slope
0.21451 ± 0.00497
Residual Sum of Squares
2.21422E-7
Pearson's r
0.99893
R-Square (COD)
0.99786
Adj. R-Square
0.99732

Figure S1. Cyclic voltammograms recorded in 0.1 M $K_3Fe(CN)_6$/0.1 M KCl at different scan rates for (A) GC, (C) SCNF-rGO, and (E) SCNF-rGO-POR electrodes. The corresponding plots of anodic peak current (I_p) versus the square root of the scan rate ($v^{1/2}$) are shown in (B), (D), and (F), respectively, confirming a diffusion-controlled electrochemical process according to the Randles–Ševčík relationship.

To select the most suitable formulation, the results from Figure S2 were analyzed. Electrochemical impedance spectroscopy (EIS) data indicate that the 1:5 rGO-SCNF composition exhibits the lowest charge-transfer resistance, enabling proper electrode behavior by minimizing ohmic effects. Consequently, subsequent characterizations and complementary analyses focused on this sample, compared to the base SCNF sample, as it was considered the most representative for the study.

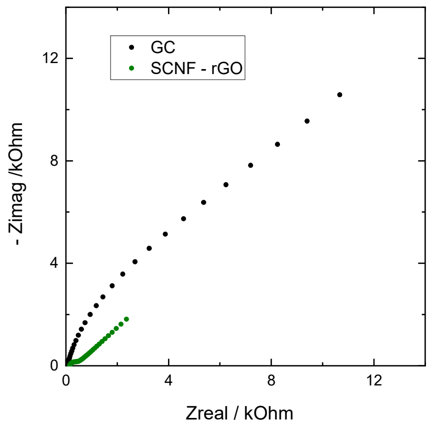

Figure S2. (a) EIS plot of the GC electrode versus the GC electrode modified with SCNF samples.

In this particular study, the polarization resistance (R_p, or charge-transfer resistance) values are so low that the resistive branch contributes minimally to the characteristic semicircle. Consequently, the Nyquist plot shows only an oblique line (Warburg-type behavior), indicating $R_p \approx 0\ \Omega$, which suggests extremely fast charge transfer. Physically, this implies that the hydrazine electrooxidation reaction is not limited by electrode kinetics; rather, the total impedance is dominated by diffusion processes of species both in the solution and within the modified film. This behavior supports the high conductivity and catalytic efficiency of the studied system. In this context, the rGO/SCNF (1:5) sample exhibits a steeper slope and lower overall impedance, translating into

enhanced ionic and electronic charge transport. For this reason, this formulation was selected as the representative system for subsequent electrochemical analyses.

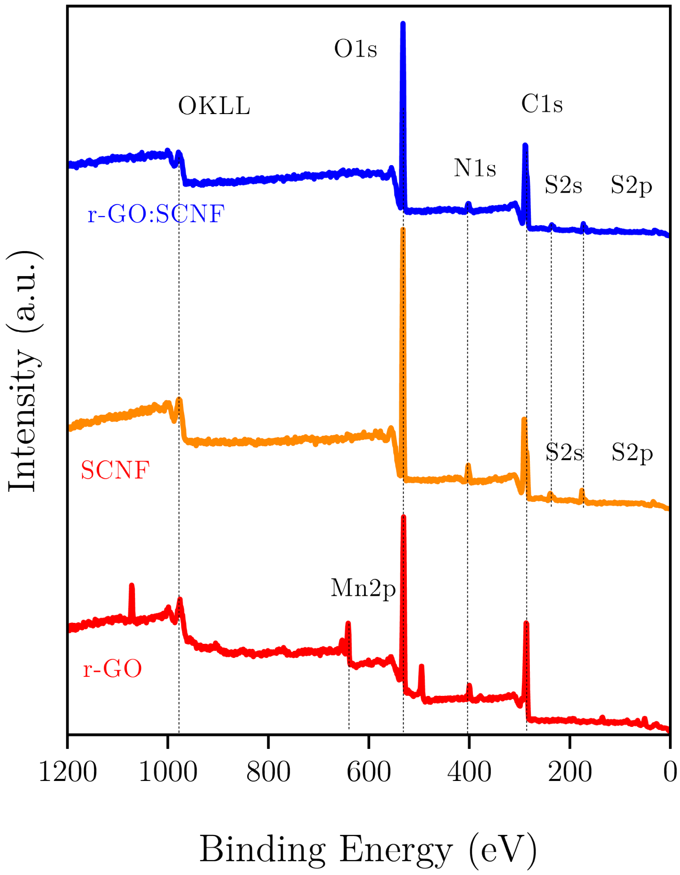

Figure S3. XPS survey spectra of r-GO (red), SCNF (orange), and the rGO:SCNF composite (blue).

The main features are labeled: O KLL Auger (~975 eV), O 1s (~532 eV), N 1s (~400 eV), C 1s (~285 eV), and the sulfur doublet S 2s/S 2p (~230 eV / ~168–170 eV). SCNF and the composite clearly display S 2s/S 2p and N 1s, consistent with sulfate esters (S^{6+}) and urea-derived carbamate/amide functionalities from the sulfamation treatment. r-GO also exhibits a Mn 2p doublet (~642/654 eV), indicating traces of Mn residuals from GO synthesis/reduction; Mn is not detected in SCNF or in the composite within XPS detection limits. All spectra were charge-referenced to the C 1s sp^2 peak at ~284.5–284.8 eV.

Table S1 presents the high-resolution XPS deconvolution of the C1s and O1s regions for r-GO, SCNF, and the r-GO/SCNF hybrid film.

The table reports the binding energy (BE), full width at half maximum (FWHM), peak area, and relative contribution of each component, enabling the identification of graphitic (sp^2) and aliphatic (sp^3) carbon, oxygenated functionalities (C–O, C=O, O–C=O), sulfate-related groups, and shake-up features. These results provide a comparative view of the surface chemical environments present in the pristine materials and their hybrid interface.

Sample	Envelope	Assignation	BE (eV)	FWHM (eV)	Area (a.u)	Relative Area (%)
r-GO	C1s	sp^2 (C=C graphitic)	284.3	2.7	1921.8	69.1
		sp^3 (C–C/C–H aliphatic)	285.1	1.2	133.9	4.8
		C–O (alcohol/ether/epoxide)	286.2	1.5	301.1	10.8
		C=O (carbonyl)	287.2	1.8	237.0	8.5
		O–C=O (carboxyl/ester)	288.3	1.7	113.9	4.1
		$\pi \rightarrow \pi^*$ (aromatic shake-up)	292.7	1.6	72.5	2.6
	O1s	C=O (carbonyl)	528.9	1.6	1012.0	21.5
		C–O (alcohol/ether/epoxide)	530.1	2.8	3154.3	67.0
		H_2O	531.4	3.0	544.7	11.6
SCNF	C1s	sp^3 (C–C/C–H aliphatic)	284.7	1.1	51.3	1.6
		C–O/C-O-C/C-O-SO_3	287.6	3.4	987.7	30.5
		O–C=O (carboxyl/ester)	289.8	2.1	1210.9	37.4
		-O-CO3	291.7	1.6	666.6	20.6
		(shake-up)	293.0	2.3	321.05	9.9
	O1s	C=O/O-C=O	531.3	2.41	4655.6	71.8
		C–O/H_2O	532.9	2.0	1828.9	28.2
r-GO/SCNF	C1s	sp^2 (C=C graphitic)	283.7	1.4	87.8	2.8
		sp^3 (C–C/C–H aliphatic)	285.1	1.6	377.9	12.1
		C–O/C-O-C	286.9	2.2	567.4	18.2
		O–C=O/C-O-SO3	289.1	1.9	1380.4	44.3
		-O-CO3	290.6	1.46	340.91	11.0
		(shake-up)	291.5	2.5	358.7	11.5
	O1s	C=O/O-C=O	531.0	2.1	2094.6	46.3
		C–O/H_2O	532.5	2.1	2426.0	53.7
